# Supplementary material for: In vivo tumor immune microenvironment phenotypes correlate with inflammation and vasculature to predict immunotherapy response
Source: Nat Commun. 2022 Sep 9;13:5312. doi: 10.1038/s41467-022-32738-7 (PMC9463451; doi:10.1038/s41467-022-32738-7)
Supplement: Supplementary file 1 — Supplementary information [file 41467_2022_32738_MOESM1_ESM.docx]

**Supplementary Information File:**

1. Supplementary Methods Table

2. Supplementary Figures

3. Source Data Information

**1. Supplementary Methods:**

| **Sr. No.** | **Antibody** | **Manufacturer:** | **CAT Number:** | **Dilution:** |
| --- | --- | --- | --- | --- |
|  | Anti-FOXP3 (236A/E7) | AbCam | ab20034 | 1:50 |
|  | Anti-PDL1 (E1L3N) | Cell Signaling | 13684S | 1:150 |
|  | Anti-CD68 (PG-M1) | Dako | M087601-2 | 1:75 |
|  | Anti-CD8 (4B11) | Bio-Rad | MCA1817 | 1:150 |
|  | Anti-PD1 (ERP4877) | AbCam | ab137132 | 1:300 |
|  | Anti-CD45, Alexa Fluor 700 (HI30) | Biolegend | 304024 | 1:200 |
|  | Anti-CD3, PE-Cy7 (OKT3) | Biolegend | 317334 | 1:200 |
|  | Anti-CD4, Pacific blue (OKT4) | Biolegend | 317424 | 1:200 |
|  | Anti-CD8, PerCP-Cy5.5 (HIT8a) | Biolegend | 300924 | 1:200 |
|  | Anti- Foxp3, APC (236A/E7) | Thermo Fisher | 17-4777-42 | 1:200 |
|  | Anti- Ki67, FITC (B56) | BD Pharmingen | 558616 | 1:200 |
|  | Anti- Gzmb, PE-Texas Red (GB11) | Thermo Fisher | GRB17 | 1:200 |
|  | Anti- CD16/CD32 Fc block (2.4G2) | BD Pharmingen | 553142 | 1:400 |
|  | Anti-CD3 (LN10) | Leica | NCL-L-CD3-565 | 1:200 |
|  | Anti-CD20 (L26) | Dako (Agilent) | M0755 | 1:3000 |
|  | Anti-CD68 (PG-M1) | Dako(Agilent) | M0876 | 1:200 |
|  | Anti-CD1a (010) | Dako(Agilent) | M3571 | 1:1000 |

Table S1. List of antibodies, manufacturer and catalog information and dilutions used in this study.

**2. Supplementary Figures**

**
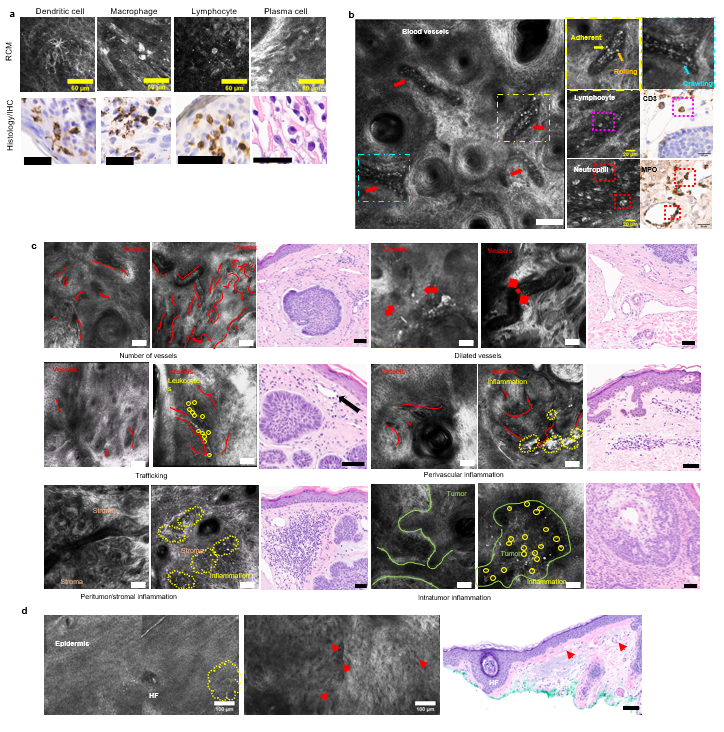
**

***Figure S1. Tumor-immune microenvironment and vasculature visualized and evaluated on RCM***

**a.** Dendritic cells, macrophages, lymphocytes and plasma cells seen on RCM are confirmed using immunohistochemistry (using anti-CD1a, anti-CD68 and anti-CD3, respectively) or H&E. Scale bar for histology: 30 μm

**b.** RCM image showing blood vessels (red arrows) and trafficking of leukocytes on the inner edge of the endothelium and individual leukocyte trafficking steps: adhesion, rolling (yellow inset) and crawling (cyan inset). Trafficking of lymphocytes and neutrophils was observed and confirmed using anti-CD3 and anti-Myeloperoxidase, respectively.

**c.** Representative examples of features assessed by manual evaluation and corresponding H&E images evaluated for RCM-histopathology correlation. For each feature, images with no/minimal feature presence and scored either 0 or 1 are shown on the left, while images with high density of features scored as 2 or 3 shown on the right (Red curve= blood vessels, diameter of vessels indicated by length of lines between diamonds; trafficking leukocytes encircled in yellow; inflammation adjacent to vessels or in stroma marked by yellow lines; intratumor immune cells encircled in yellow). Black arrow in H&E image within the trafficking panel indicates “stuck” leukocytes indicating trafficking.

**d.** Representative mosaic showing normal epidermis and hair follicle in the perilesional area with a dendritic cell (yellow) in the left image, mosaic showing collagen fibers and small blood vessels (red arrows) in the dermis in the middle image. Corresponding H&E image from the perilesional area showing normal skin, hair follicle and thin blood vessels with few scattered immune cells in dermis.

Scale bar for RCM: 100 μm, H&E: 60 μm unless stated otherwise. RCM images are representative images for each feature selected by experts after reviewing all images.


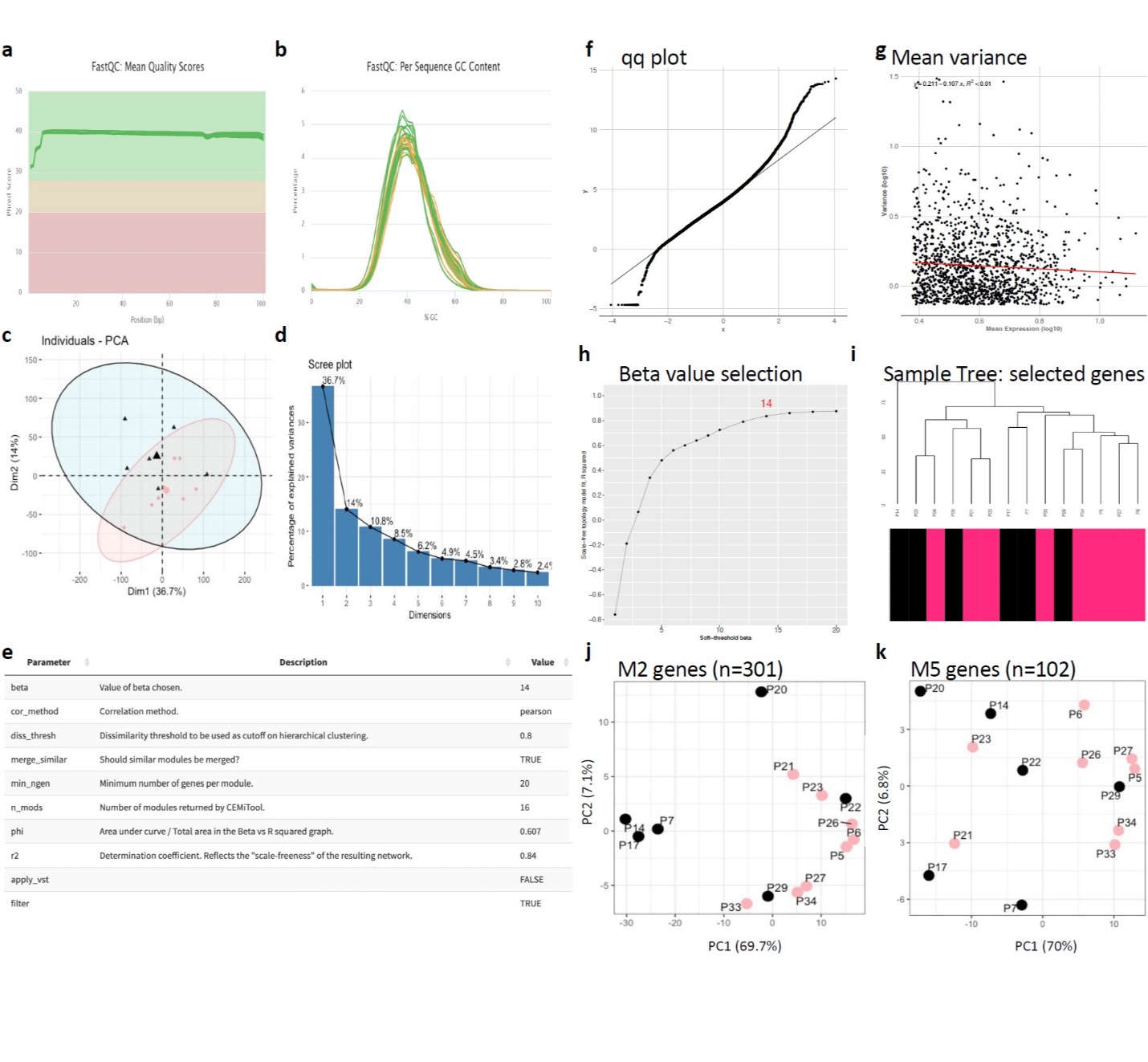


***Figure S2. QC and feature selection in transcriptomic analysis***

***a.*** Mean quality score of bases at each position in sequencing read. Mean phred score > 28 indicates good quality (green).

**b.** GC content across all reads forms a normal distribution with peak of curve indicating mean GC content at ~40%.

**c&d.** PCA of full transcript abundance for 14 BCC samples showing sample groups evenly distributed along PC1 (36.7% variance) while PC2 (14% variance) captures variation in transcript abundance between RCM phenotypes (Inflam^HIGH^Vasc^HIGH^= pink, Inflam^LOW^Vasc^HIGH^= black). Confidence ellipses drawn around categories (CI = 95%, group means indicated by larger size points near center of graph) fail to separate RCM phenotypes.

**e.** Table of parameters used to identify gene co-expression modules using CEMiTool.

**f.** Quantile-quantile plot shows normal distribution of genes expressed across 14 BCC samples, allowing for Pearson method to be chosen for correlation of genes using CEMiTool.

**g**. Scatterplot of the mean expression of all genes by the variance of expression. No strong relationship is identified, r^2^ < 0.01 (red line), therefore no variance stabilizing transformation was applied to the data using CEMiTool.

**h.** Beta by r^2^ plot explains the selection of the soft-thresholding parameter Beta (shown in red, beta = 14). Threshold for R^2^  > 0.8 using CEMiTool.

**i.** Sample clustering tree for selected genes (n=1,379, p-value < 0.1) by CEMiTool used in identifying gene co-expression modules. Two clusters of closely related samples are revealed, although the relationship is not explained by RCM phenotype (one outlier sample, P14).

**j&k.** PCA of transcript abundance from genes comprising M2 (j) and M5 (k). PC1 shows major source of variation in transcript abundance is RCM phenotype (Inflam^HIGH^Vasc^HIGH^= pink, Inflam^LOW^Vasc^HIGH^= black) for M2, yet less resolved in M5. Source data are provided as a Source Data file.


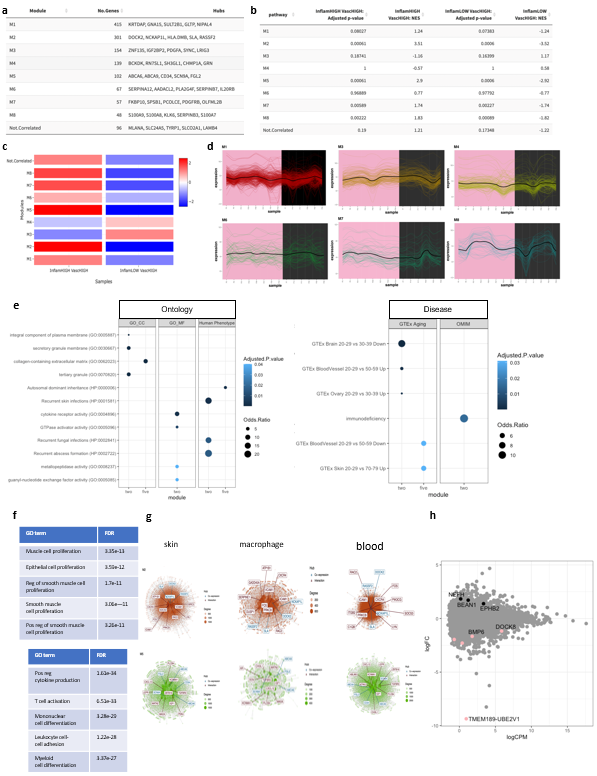


***Figure S3. Transcriptomic analysis using co-expression modules, gene ontology and gene regulatory networks.***

**a.** Summary of modules identified by CEMiTool indicating number of correlated genes in each module along with top 5 genes with the highest connectivity in each module (hubs).

**b.** Table for module enrichment in RCM phenotypes. Genes within each module are provided as the gene set and the median z score values of each phenotype as rank. Enrichment scores are normalized to the number of genes in the module.

**c.** Activity of each module in RCM phenotype classes are displayed as heatmap. The intensity of the color corresponds to the normalized enrichment score (NES), red indicates high activity and blue is low activity.

**d.** Profile plots of genes in modules showing no enrichment in RCM phenotype groups; M1 (415 genes), M3 (154 genes), M4 (139 genes), M6 (67 genes), M7 (57 genes), and M8 (48 genes). Colored lines show expression levels for individual genes and the black line represents mean expression of all genes in the module (expression plotted on y axis = log_2_cpm). Individual patient samples are displayed on X axis and colored by RCM phenotype (Inflam^HIGH^Vasc^HIGH^= pink, Inflam^LOW^Vasc^HIGH^= black).

**e.** Enrichment (adj. p-value < 0.05, with multiple testing correction using BH adjusted pvalue) of M2 and M5 for GO terms (CC = Cellular Component , MF = Molecular Function, and Human Phenotype) and human disease (GTEx Aging and OMIM).

**f.** GO enrichment for hub genes along with interacting partners participating in local functional gene regulatory network in T lymphocytes (curated by TissueNexus) are shown (Top 5 terms, adj. pvalue < 0.05).

**g.** Gene networks of M2 and M5 in skin, macrophage and blood. Top 10 most connected genes (Hub) in network are labeled (interaction = red). Module hub genes identified in network are indicated in blue (co-expression). Nodes indicate genes (size is proportional to degree) and edges represent connections to genes in network. Module hub genes persist as participating genes in functional gene regulatory networks across related cell types and tissue involved in BCC pathogenicity, with largely overlapping network hub genes.

**h.** MA plot highlighting genes differentially expressed between RCM phenotype groups in BCC samples (pairwise comparison, FDR < 0.05). Overall, the majority of genes do not show a significant difference between RCM phenotypes (gray n=21,074). Among the genes significantly upregulated in the low inflammatory group (3 genes, black), *EPHB2* belongs to M2 and *NEFH* overlaps with immune genes identified by CIBERSORTx. Five genes were significantly upregulated in the high inflammatory group (pink) and showed overlap with *JPH2* in immune genes identified by CIBERSORTx. Source data are provided as a Source Data file.


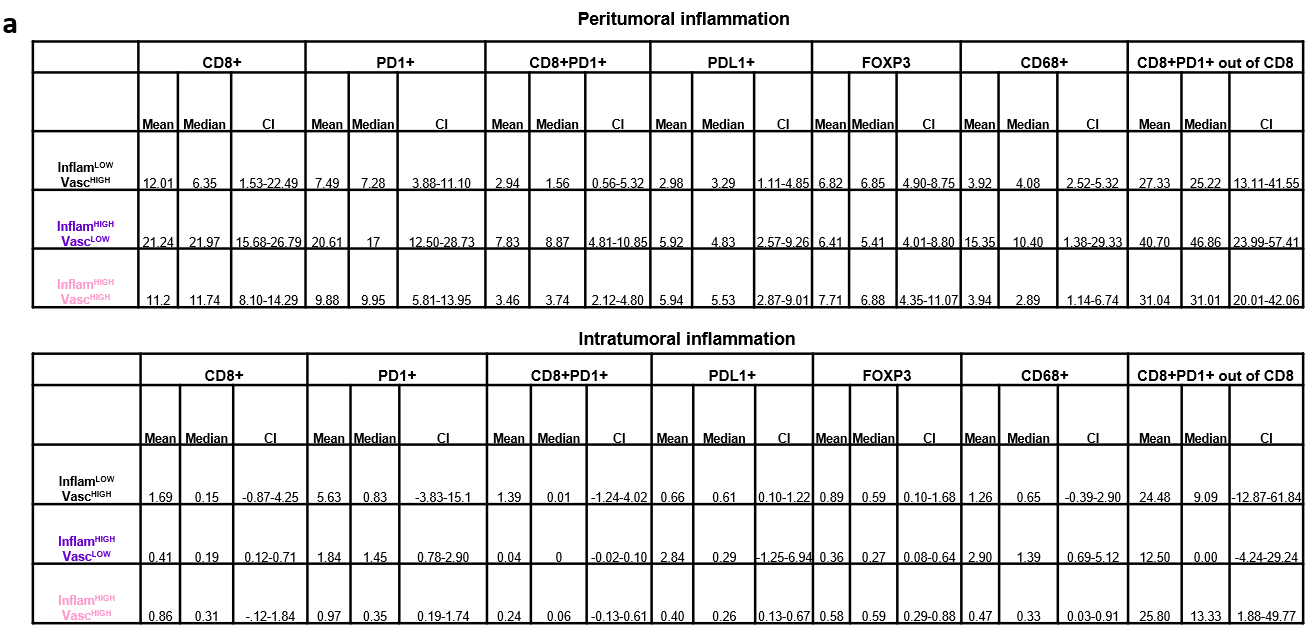

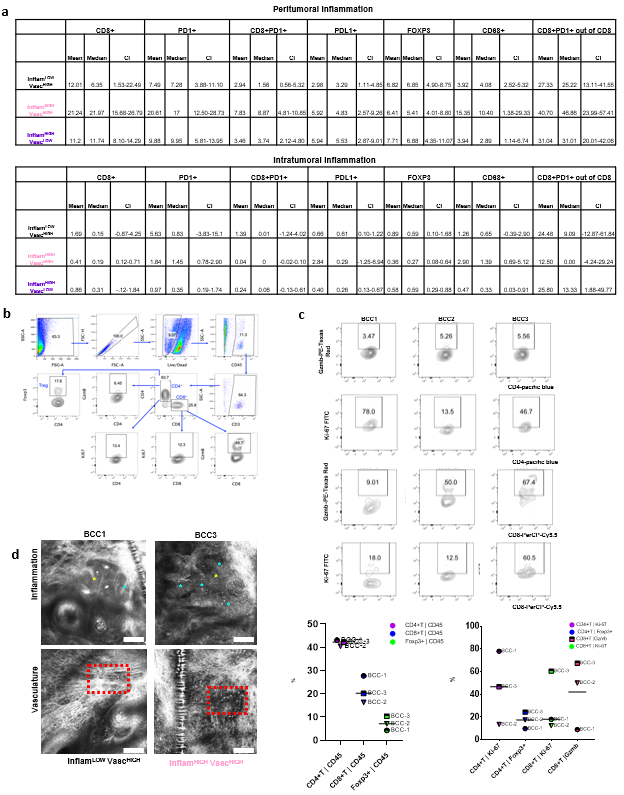


***Figure S4. Immunophenotyping correlates with RCM phenotypes***

**a.** Multiplexed IF analysis (CD8^+^, Foxp3, CD68^+^, PD-1^+^ and PD-L1^+^) on n=24 BCC specimens quantified in peritumoral and intratumoral areas. Average of 1-12 ROIs were analyzed to compute mean, median and confidence intervals for each of the three groups: Inflam^LOW^Vasc^HIGH^ (black), Inflam^HIGH^Vasc^LOW^ (purple) and Inflam^HIGH^Vasc^HIGH^ (pink). Source data are provided as a Source Data file.

**b.** Gating strategy for selecting the CD4^+^ and CD8^+^ subpopulations from BCC tumor tissues (n=3)

**c.** Contour and dot plots showing the fraction of Ki-67^+^ and GzmB^+^ CD4^+^ and CD8^+^ cells in n= 3 BCC patients. Higher activated and proliferating populations of CD8^+^ T cells, higher FOXP3 were seen in BCC3 (Inflam^HIGH^Vasc^HIGH^) while BCC1 (Inflam^LOW^Vasc^HIGH^) showed higher proliferating CD4^+^ T cells.

**d.** Representative RCM images from BCC1 (Inflam^LOW^Vasc^HIGH^) and BCC3 (Inflam^HIGH^Vasc^HIGH^) patients highlighting the differences in intratumoral inflammation, trafficking and vasculature (yellow asterisk- tumor, blue asterisk-immune cells, red box-vessels and trafficking) (Scale bar: 100 μm)


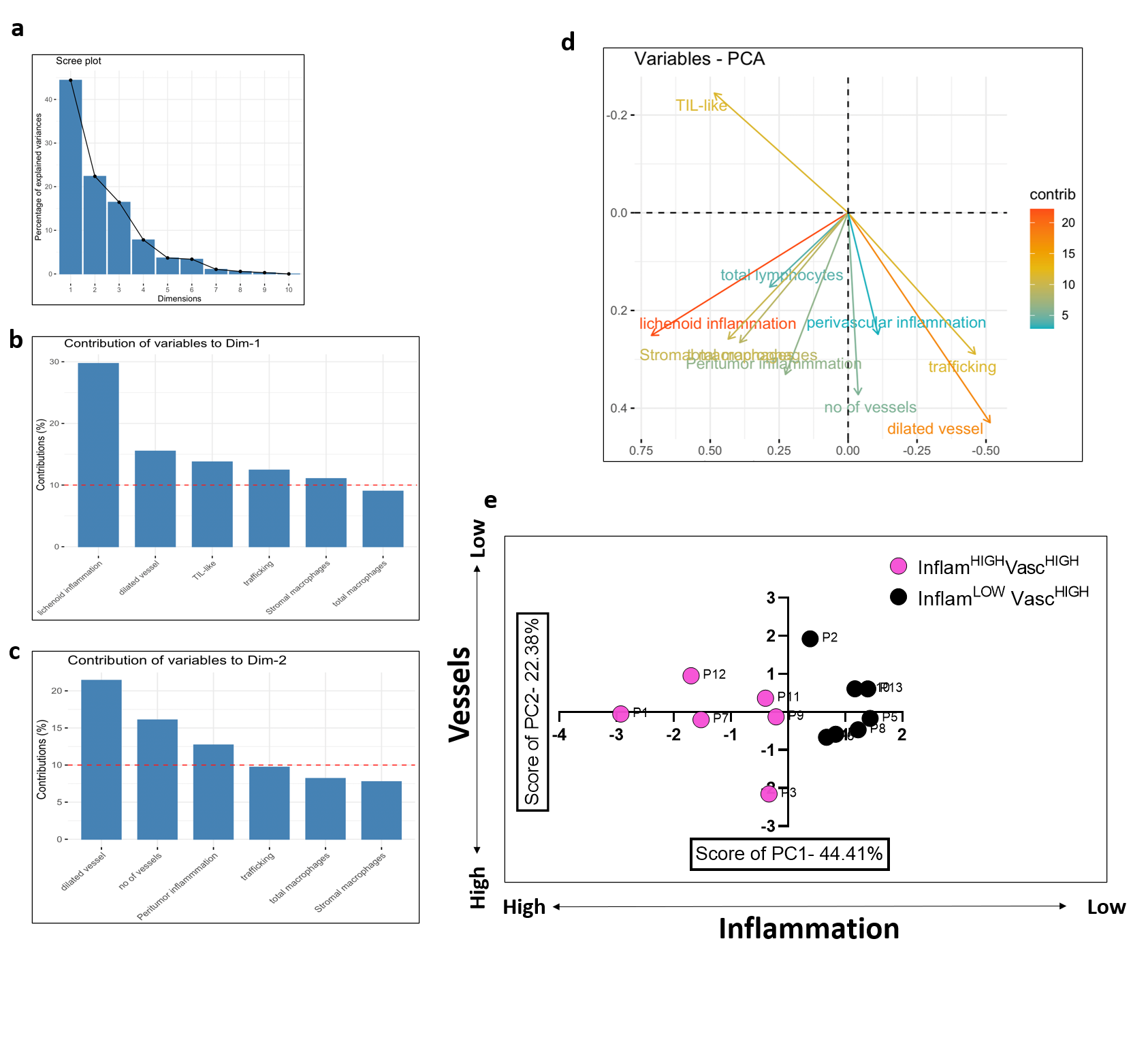


***Figure S5. TiME phenotyping group assignment in melanoma patients using principal component analysis (PCA)***

**a.** Scree plot depicting the percentage contribution to variance for each PC. Top 2 PCs encompassing ~64% variance were used for phenotype group assignments.

**b.** Lichenoid features (inflammation feature) had the highest contribution to dimension 1 or PC1 (~30%), almost twice that of the dilated vessels (~15%) and TIL-like cells (~14%) . Thus, we assigned PC1 predominantly to inflammation.

**c.** Dilated vessels (~21%) and number of vessels (~16%) were the top 2 contributors to the dimension 2 or PC2, followed by peritumor inflammation (~12.5%). Thus, we assigned PC2 predominantly to vasculature.

**d.** Biplot depicting contribution of each variable in PC1 and PC2

**e.** Scatter plot for PC1 vs PC2 produces two clusters that were assigned to Inflam^HIGH^Vasc^HIGH^ (pink) or Inflam^LOW^Vasc^HIGH^ (black) phenotype since PC1 classifies phenotypes based on mainly inflammation while PC2 classified phenotypes mainly based on vascular features. Thus, samples along PC1 cluster mainly based on inflammation **(**low = black, high = pink) and samples distributed along PC2 cluster based on vasculature **(**high = pink and black). Since the clusters were distribution across similar co-ordinates along PC2, we labeled both clusters as Vasc^HIGH^. Source data are provided as a Source Data file.


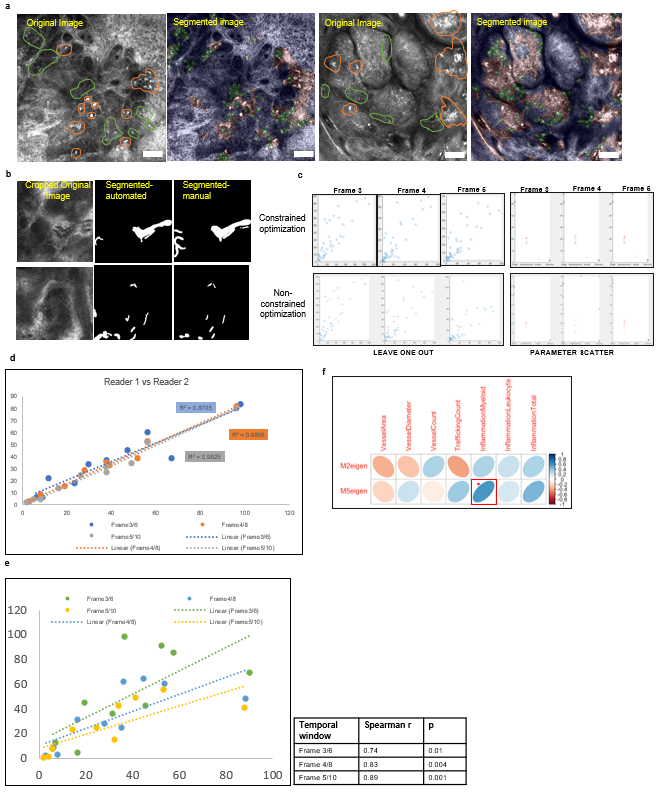


***Figure S6. Automated quantification of TiME and vasculature features on RCM images and correlation with TiME features***

**a.** Representative RCM images (original and segmented image) showing segmentation of leukocyte-like cells (green), and dendritic and macrophages (orange) using a UNet model trained on 1026 images (926-training, 100-validation) yielding a Dice coefficient of 0.72. This model was used to segment immune cell density on 652 independent images used in TiME analysis. Scale bar: 100 μm

**b.** Exemplar RCM video frames (original, automated segmented, manually segmented) showing segmentation of blood vessels from single field-of-view RCM videos. Comparison of manual and automated segmentations on 23 videos yielded a median Dice coefficient of 0.52 (0.29-0.78). This segmentation was used to derive vasculature features such as blood vessel diameter, area and number of vessels used in TiME analysis.

**c.** Summary of constrained and non-constrained optimization of parameters for leukocyte trafficking of: leave-one out and parameter scatter (angle, displacement, length and intensity) are presented.

**d.** Agreement between manual counts by two independent readers on 10 videos.

e. High Spearman correlation (0.74-0.89) observed for average manual reader count and automated counts for all 3 temporal windows. The 3 frame (0.6 s) temporal window was selected for TiME analysis to include both faster (rolling) and slower (crawling) trafficking events.

**f.** Spearman correlation for eigengene values for gene co-expression modules enriched in RCM phenotypes, M2 and M5, with TiME features show significant correlation for M5 with total myeloid cells (outlined in red; *pvalue < 0.05).

Representative examples of RCM original and segmented images are shown and selected from the pool of images acquired from each patient. Source data are provided as a Source Data file.


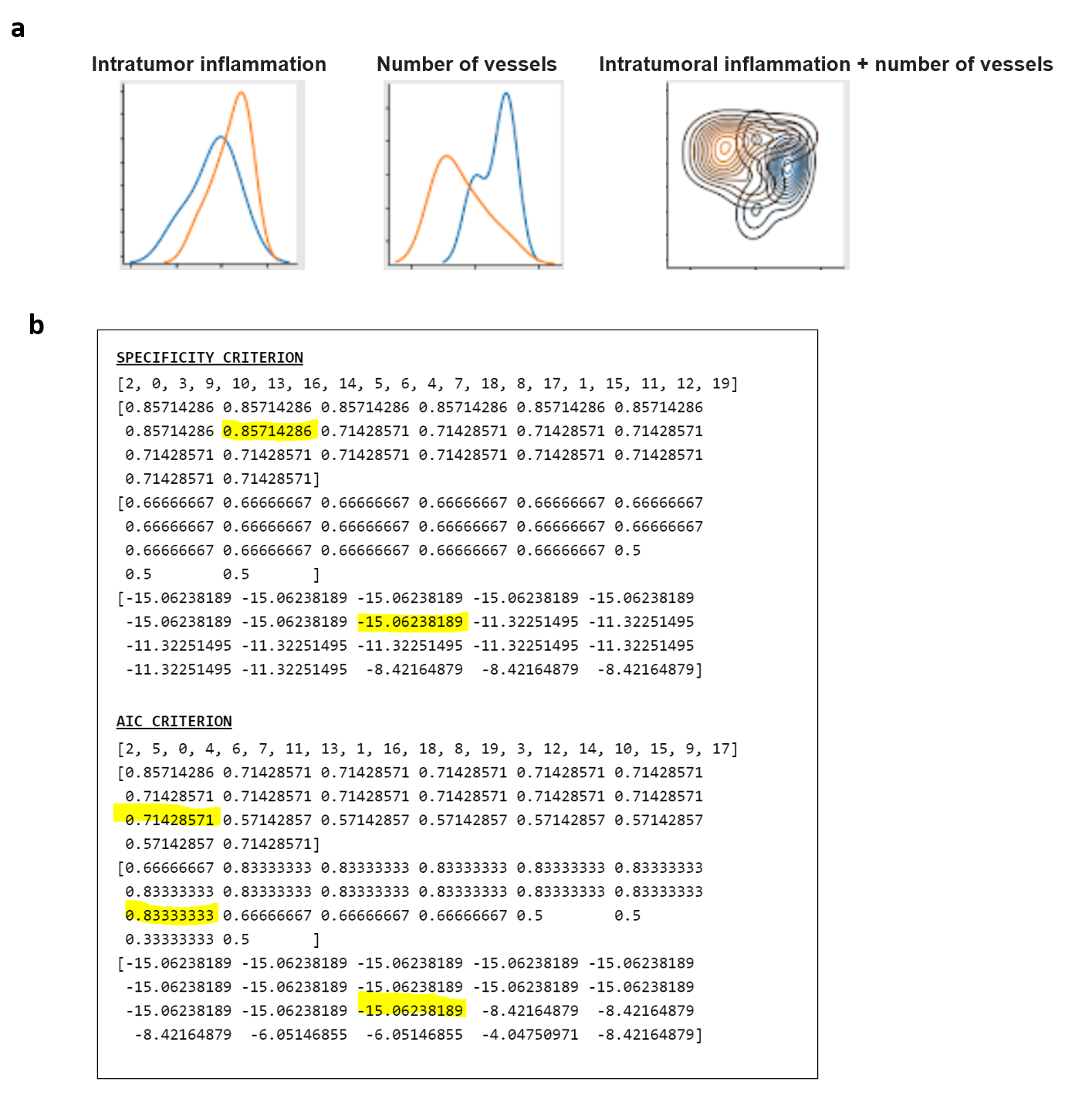


***Figure S7. Modeling of imiquimod response highlights importance of stromal features in improving predictive power of linear regression models.***

**a.** Linear separability curves for intratumor inflammation, number of vessels which were predicted as priority labels in AIC and specificity prioritizing models. Combined plot for stromal vessels + intratumoral inflammation shows maximum separability (orange- responders, blue-non-responders).

**b.** Models based on prioritizing Specificity or AIC as criteria for selecting features associated with response in linear regression models. For specificity, sequential modeling using 13 factors gave optimum performance while for AIC models the best performance was seen at 8 features.

**3. Source Data Information**

Source data has been provided for Figures 2, 3 4, 5, 6 and 7 (including Figures S3, S4, S5, S6 and S7) and included in a zipped folder.

**Figure 2:**

- Excel sheet containing RCM evaluation from 27 BCC lesions used for HCPC and PCA in figure 2.

**Figure 3:**

- Cpmnormlog2_filtergeneid_inputcemi.txt file was used as input for generating gene expression modules using CEMiTool. Supplemental Figure 2c-e and Figure 3a-e as well as Supplemental Figure 3a-f were generated using this input gene expression data.
- Modulegenes.tsv is a list of genes and respective module assignment resulting from CEMiTool analysis
- Zipped folder for GO enrichment using enrichr for module 2 and 5 associated with figure 3c and S3e
- CIBERSORTx_Job12_Results.xlsx CIBERSORTx output estimating cell proportions for each sample used to generate plots for Figure 3f and h.
- LM22.txt reports genes used to estimate cell proportions resulting from CIBERSORTx. Differential expression of these transcripts across samples were used to generate plot in Figure 3g.
- Zipped files in TissueNexusInteraction were used to generate module gene interactions for specific tissues/cell type (blood.txt, macrophage.txt, skin.txt, t_lymphocyte.txt) displayed in Figure 3d and Supplemental Figure S3g. Gene expression for module hub genes along with network hub genes (intermod2hubtidy.txt and intermod5hubtidy.txt) were shown in Figure 3e.

**Supplemental Figure 3:**

- edgeRDEGfullbulk_pairwiseresults.txt files reports the results from pairwise comparison of differential gene expression analysis using edgeR. logFC, average logCPM expression, PValue, FDR, and gene ensemble id as well as gene symbol are reported. Supplemental Figure 3g MA plot was generated using this output.

**Figure 4 and Supplemental Figure 4:**

- Sheet 4a contains the multiplexed IF analysis values, including raw counts and calculated positive cell counts in both intratumoral and peritumoral regions in BCCs that were used to generate column scatter plots showing distribution of cells across the three phenotypes in figure 4a and supplementary figure S4a
- Sheet 4b contains the calculated CD3^+^ CD20^+^ positive and TLS areas in BCC specimens that were used to generate the column scatter plots showing distribution of cell and TLS positivity across the three phenotypes in figure 4b

**Figure 5 and Supplemental Figure 5:**

- Sheet 5a contains RCM evaluation from 13 melanoma lesions used for HCPC and PCA analysis in figure 5a
- Sheet 5b contains calculated CD3^+^ T-cell area positivity and TLS area in melanoma specimens that were that were used to generate the column scatter plots showing distribution of cell and TLS positivity across the two phenotypes in figure 5b

**Figure 6:**

- Sheet 6a contains the quantified RCM values for vessel diameter, count and area, vessel trafficking counts, total inflammation density and only leukocyte-like density along with corresponding gene expression values used for correlation and plotted in figure 6a. The RCM values were also used in correlation analysis with module eigenvalues in figure 6b.
- Sheet 6b the eigen value for each module from CEMiTool which was used to correlate with RCM TiME traits; module 5 sig correlated with infiltrating myeloid shown in Figure 6b.

**Supplemental Figure 6:**

- Sheet S6 reports results from Spearman correlation of M2 and M5 eigengene values with RCM TiME phenotypes. Correlation and pvalue for each relationship is reported and represented as correlation matrix in Figure S6f and scatterplot of M5 eigenvalue against infiltrating myeloid cells in Figure 6b

**Figure 7 and Supplemental Figure 7:**

- Sheet 7a lists detailed RCM evaluation for imiquimod responders (R) and non-responders (NR) used to create HCPC clusters in figure 7a and heatmap, plots in figure 7c. These evaluations were used for linear regression modeling in figure S7.
- Sheet 7b lists RCM evaluation for key features from original analysis on 27 BCC lesions and 13 imiquimod treated lesions to predict phenotype in figure 7b
