# Supplementary material for: In vivo tumor immune microenvironment phenotypes correlate with inflammation and vasculature to predict immunotherapy response
Source: Nat Commun. 2022 Sep 9;13:5312. doi: 10.1038/s41467-022-32738-7 (PMC9463451; doi:10.1038/s41467-022-32738-7)
Supplement: Supplementary file 2 — Reporting Summary [file 41467_2022_32738_MOESM2_ESM.pdf]

nature portfolio

Corresponding author(s): Aditi Sahu
Milind Rajadhyaksha

Last updated by author(s): Jul 4, 2022

Reporting Summary

Nature Portfolio wishes to improve the reproducibility of the work that we publish. This form provides structure for consistency and transparency in reporting. For further information on Nature Portfolio policies, see our Editorial Policies and the Editorial Policy Checklist.

Statistics

For all statistical analyses, confirm that the following items are present in the figure legend, table legend, main text, or Methods section.

n/a	Confirmed	
☐	☒	The exact sample size (*n*) for each experimental group/condition, given as a discrete number and unit of measurement
☐	☒	A statement on whether measurements were taken from distinct samples or whether the same sample was measured repeatedly
☐	☒	The statistical test(s) used AND whether they are one- or two-sided<br>*Only common tests should be described solely by name; describe more complex techniques in the Methods section.*
☐	☒	A description of all covariates tested
☐	☒	A description of any assumptions or corrections, such as tests of normality and adjustment for multiple comparisons
☐	☒	A full description of the statistical parameters including central tendency (e.g. means) or other basic estimates (e.g. regression coefficient) AND variation (e.g. standard deviation) or associated estimates of uncertainty (e.g. confidence intervals)
☐	☒	For null hypothesis testing, the test statistic (e.g. *F*, *t*, *r*) with confidence intervals, effect sizes, degrees of freedom and *P* value noted<br>*Give P values as exact values whenever suitable.*
☒	☐	For Bayesian analysis, information on the choice of priors and Markov chain Monte Carlo settings
☒	☐	For hierarchical and complex designs, identification of the appropriate level for tests and full reporting of outcomes
☐	☒	Estimates of effect sizes (e.g. Cohen's *d*, Pearson's *r*), indicating how they were calculated

Our web collection on statistics for biologists contains articles on many of the points above.

Software and code

Policy information about availability of computer code

Data collection	VivaScan v10.0 (Caliber Imaging and Diagnostics, Rochester, NY, USA)
Data analysis	Fiji/ImageJ 1.53c (which includes Linear Alignment with Sift Plugin and TrackMate v6.0.3)<br>Aperio ImageScope version 12.4.3.5008 (Leica Biosystems, Wetzlar, Germany)<br>Matlab R2019b (The MathWorks, Inc., Natick, MA, USA)<br>GraphPad Prism 9.0 (Dotmatics, San Diego, CA, USA)<br>Vectra Polaris Automated Quantitative Pathology Imaging System (Akoya 514 Biosciences, Marlborough, MA, USA)<br>HALO version 2.3 (Indica Labs, Albuquerque, NM, USA)<br>R version 3.6.3<br>FlowJo version 10.5.3.<br>Custom image and data analysis scripts for Fiji and Matlab (https://github.com/mskccmccf/TiME-analysis)

For manuscripts utilizing custom algorithms or software that are central to the research but not yet described in published literature, software must be made available to editors and reviewers. We strongly encourage code deposition in a community repository (e.g. GitHub). See the Nature Portfolio guidelines for submitting code & software for further information.

March 2021

Data

Policy information about availability of data

All manuscripts must include a data availability statement. This statement should provide the following information, where applicable:

- Accession codes, unique identifiers, or web links for publicly available datasets
- A description of any restrictions on data availability
- For clinical datasets or third party data, please ensure that the statement adheres to our policy

The RNA-expression datasets generated during this study have been made available on gene expression omnibus (GEO). The GEO ID is GSE181037. Publicly available datasets used in this study include MsigDB (https://www.gsea-msigdb.org/gsea/msigdb/), gene ontology (http://geneontology.org/), GTex (https://gtexportal.org/home/) and OMIM repository (https://www.ncbi.nlm.nih.gov/omim). Source data are provided with this paper. Raw images will be freely available upon request. As per MSKCC guidelines, a data sharing agreement will have to be necessarily set up with requesting colleagues and their institutions.

Human research participants

Policy information about studies involving human research participants and Sex and Gender in Research.

Reporting on sex and gender	Only sex was included in the study design and data analysis. These values were derived from patient charts in the electronic medical record. The effect of sex on BCC and melanoma phenotyping was analyzed in the manuscript - no correlation was found, suggesting that sex had no or minimal influence on TiME phenotyping. Disaggregated sex and gender data was not collected, since it was not pertinent to the study design.
Population characteristics	Dermatology Service at Memorial Sloan Kettering Cancer Center (MSKCC) specializes in skin cancer management. Skin lesions seen at MSKCC include keratinocytic cancers such as basal cell carcinoma (BCC), squamous cell carcinoma (SCC) and actinic keratosis(AK), melanocytic cancer such as melanoma, and drug- or therapy-induced skin rashes. Patients visiting Dermatology service for consultation and treatment of these skin lesions were recruited in this study. The age range of patients was 30-88 years, and the male:female ratio was 1.28:1. Several patients also had previous history of skin cancer or other solid cancer due to the patient population normally seen at this tertiary cancer center. Sun exposure, age and sex were the major patient covariates used in the analysis.
Recruitment	Patients visiting the Dermatology Service at MSKCC and presenting with previously biopsied or clinically suspected basal cell carcinoma (BCC), squamous cell carcinoma (SCC), actinic keratosis (AK), melanoma, or skin rash were prospectively recruited to avoid any selection bias. Of these, only lesions without any prior biopsy or treatment were 'selected' for phenotyping analysis. This pre-selection was determined to avoid influence of extraneous inflammatory processes such as wound healing on TiME phenotyping.
Ethics oversight	Memorial Sloan Kettering Cancer Center- Institutional Review Board approved the study. Written informed consent was obtained from all participants recruited under the active IRB protocols IRB#99-099 and IRB#21-019. All research was performed strictly in accordance with the Declaration of Helsinki and relevant guidelines and regulations.

Note that full information on the approval of the study protocol must also be provided in the manuscript.

Field-specific reporting

Please select the one below that is the best fit for your research. If you are not sure, read the appropriate sections before making your selection.

☒ Life sciences ☐ Behavioural & social sciences ☐ Ecological, evolutionary & environmental sciences

For a reference copy of the document with all sections, see nature.com/documents/nr-reporting-summary-flat.pdf

Life sciences study design

All studies must disclose on these points even when the disclosure is negative.

Sample size	No sample size calculations were performed due to the preliminary nature of this study.<br>Patients that fulfilled the inclusion criteria were prospectively accrued (defined in methods).<br>The ultimate sample size was based on patient availability and logistical constraints.
Data exclusions	Data from 1 patient was excluded due to motion blur during image acquisition.
Replication	In this cross-sectional observational study, imaging was performed on 118 patients with different cutaneous cancers and inflammatory rashes in real-time. Since these lesions are normally biopsied after imaging procedure as part of standard of care, re-imaging to verify reproducibility of features is not feasible. As an alternative, agreement statistics between readers confirmed the reproducibility of RCM features, which were also subsequently verified on histopathology.
Randomization	Our study did not involve any intervention and was purely an observational research study (i. e., not a trial). Thus, randomization was not

relevant to the study design.

Blinding

This study was an observational research study and not a clinical trial. The group or phenotype allocation was performed purely through unsupervised clustering algorithms that only interpret the input data and find natural groups or clusters based on inherent features, rather than any external inputs or labels. This approach eliminated the scope of bias in the analysis and therefore blinding was not done in this study.

Reporting for specific materials, systems and methods

We require information from authors about some types of materials, experimental systems and methods used in many studies. Here, indicate whether each material, system or method listed is relevant to your study. If you are not sure if a list item applies to your research, read the appropriate section before selecting a response.

Materials & experimental systems

n/a	Involved in the study	
☐	☒	Antibodies
☒	☐	Eukaryotic cell lines
☒	☐	Palaeontology and archaeology
☒	☐	Animals and other organisms
☐	☒	Clinical data
☒	☐	Dual use research of concern

Methods

n/a	Involved in the study	
☒	☐	ChIP-seq
☐	☒	Flow cytometry
☒	☐	MRI-based neuroimaging

Antibodies

Antibodies used

CD3(LN10) from Leica, catalog# NCL-L-CD3-565
CD20(L26) from Dako (Agilent), catalog# M0755
CD68(PG-M1), from Dako (Agilent), catalog# M0876
CD1a (010) from Dako (Agilent), catalog# M3571
FOXP3 (236A/E7) from AbCam, catalog# ab20034
PDL1 (E1L3N) from Cell Signaling, catalog# 13684S
CD68 (PG-M1) from Dako, catalog# M087601-2
CD8 (4B11) from Bio-Rad, catalog# MCA1817
PD1 (ERP4877) from AbCam, catalog# ab137132
CD45, Alexa Fluor 700, clone HI30, Biolegend, catalog# 304024
CD3, PE-Cy7, clone OKT3, Biolegend, catalog# 317334
CD4, Pacific blue, clone OKT4, Biolegend, catalog# 317424
CD8, PerCP-Cy5.5, clone HIT8a, Biolegend, catalog# 300924
Foxp3, APC, clone 236A/E7, Thermo Fisher, catalog # 17-4777-42
Ki67, FITC, clone B56, BD Pharmingen, catalog# 558616
Gzmb, PE-Texas Red, clone GB11, Thermo Fisher, catalog# GRB17
CD16/CD32 Fc block, clone2.4G2, BD Pharmingen, catalog# 553142

Validation

The antibodies used for flow cytometry were QC for flow cytometry applications. The antibodies CD45, CD3, CD4, CD8, Foxp3, Ki-67 and Gzmb was developed, and showed primary reactivity for human samples while CD16/32 Fc block was developed, and showed primary reactivity for mouse. The antibodies used for immunohistochemistry (IHC) were optimized and recommended for IHC with validated protocols on the manufacturer's website. Single-plex IHC antibody assays were titrated based on the manufacturer's recommendations. Normal human skin and normal tonsil were used for titration of CD3, CD20, CD68 and CD1a, and duplex staining conditions for CD3/CD20 were validated on basal cell carcinoma FFPE tissue sections. For the multiplexed IF, single and dual IHC, extensively validated antibodies (see clone and reference publications below) were used. The multiplex assay was further validated on human tonsil FFPE tissues.
CD45, Alexa Fluor 700, clone HI30, Biolegend, catalog# 304024
DOI: 10.1016/j.cell.2018.11.021
DOI: 10.1016/j.cell.2019.09.035
CD3, PE-Cy7, clone OKT3, Biolegend, catalog# 317334
DOI: 10.1016/j.omto.2019.12.003
DOI: 10.1016/j.cmet.2017.05.018
CD4, Pacific blue, clone OKT4, Biolegend, catalog# 317424
DOI: 10.1016/j.ccell.2017.11.001
DOI: 10.1016/j.cell.2020.09.054
CD8, PerCP-Cy5.5, clone HIT8a, Biolegend, catalog# 300924
DOI: 10.1038/s41586-018-0792-9
DOI: 10.1016/j.immuni.2019.01.001
Foxp3, APC, clone 236A/E7, Thermo Fisher, catalog # 17-4777-42
DOI: 10.1038/s41591-018-0070-2
DOI: 10.1038/s41423-019-0316-z
Ki67, FITC, clone B56, BD Pharmingen, catalog# 558616

DOI: 10.1038/nature12207
DOI: 10.1038/ncomms8158
Gzmb, PE-Texas Red, clone GB11, Thermo Fisher, catalog# GRB17
DOI: 10.1016/j.cell.2020.11.007
DOI: 10.1136/jitc-2020-001355
CD16/CD32 Fc block, clone2.4G2, BD Pharmingen, catalog# 553142
DOI: 10.1038/324372a0
DOI: 10.1126/science.2946078
CD3, clone LN10, Leica, catalog# NCL-L-CD3-565
DOI: 10.1111/j.1365-2559.2011.04097.x
DOI: 10.1172/JCI129965
CD20, clone L26, Dako (Agilent), catalog# M0755
DOI: 10.1073/pnas.2007206117
DOI: 10.1038/s41467-022-29040-x
CD68, clone PG-M1, Dako (Agilent), catalog# M0876
DOI: 10.1038/s41586-021-03710-0
DOI: 10.1038/s41598-020-67987-3
CD1a, clone 010, Dako (Agilent), catalog# M3571
DOI: 10.1016/j.jconrel.2019.02.028
DOI: 10.1111/pin.13044
FOXP3, clone 236A/E7, Abcam, catalog# ab20034
DOI: 10.4049/jimmunol.177.9.5852
DOI: 10.1038/nature24462
PDL1, clone E1L3N, Cell Signaling, catalog#13684S
DOI: 10.1371/journal.pone.0136023
DOI: 10.1186/s12894-016-0195-x
CD68, clone PG-M1, Dako, catalog# M087601-2
DOI: 10.1038/modpathol.2017.144
DOI: 10.1111/bjd.13628
CD8, clone 4B11, Bio-Rad, catalog# MCA1817
DOI: 10.1002/ibd.20023
DOI: 10.1111/jdv.13989
PD1, clone ERP4877, AbCam, catalog# ab137132
DOI: 10.1007/s10120-014-0440-5
DOI: 10.1038/ncomms12335

Clinical data

Policy information about clinical studies
All manuscripts should comply with the ICMJE guidelines for publication of clinical research and a completed CONSORT checklist must be included with all submissions.

Clinical trial registration	NCT00588315
Study protocol	clinicaltrials.gov
Data collection	Dermatology Service, Memorial Sloan Kettering Cancer Center. Patients were recruited between November 2018 - November 2021
Outcomes	The primary outcome was tumor-immune microenvironment phenotyping for patient stratification. The secondary outcomes were correlation of TiME phenotyping with biological markers, and response to treatment.

Flow Cytometry

Plots

Confirm that:

- [x] The axis labels state the marker and fluorochrome used (e.g. CD4-FITC).
- [x] The axis scales are clearly visible. Include numbers along axes only for bottom left plot of group (a 'group' is an analysis of identical markers).
- [x] All plots are contour plots with outliers or pseudocolor plots.
- [x] A numerical value for number of cells or percentage (with statistics) is provided.

Methodology

Sample preparation	Skin lesions (3-mm) freshly excised from human subjects during a surgical procedure were used for flow cytometry for T-cell phenotyping. Briefly, the tumors were digested with Liberase (1.67 Wünsch U/ml) and DNase I (0.2 mg/ml) for 30 min at 37° C. After dissociation of the tumor tissue in the Miltenyi GentleMACS™ Dissociator, cell suspensions were incubated with red blood cell lysis buffer on ice for 5 min and then quenched with cold PBS. The cell pellets were resuspended with MACS buffer (Miltenyi Biotec) to generate single cell suspension and then filtered through 70 µm nylon mesh prior to FACS analysis.
Instrument	Cytek Aurora (Cytek Biosciences)
Software	FlowJo version 10.5.3
Cell population abundance	No sorting performed.
Gating strategy	Cells were gated based on FSC/SSC, followed by live-dead staining to remove dead cells, gated again for CD45 fraction (immune cells) and then for CD3. After that, individual CD4, CD8 markers and their functional (Gzmb, Foxp3) or proliferation (Ki-67) states were determined.

☒ Tick this box to confirm that a figure exemplifying the gating strategy is provided in the Supplementary Information.
