# Supplementary material for: In vivo tumor immune microenvironment phenotypes correlate with inflammation and vasculature to predict immunotherapy response
Source: Nat Commun. 2022 Sep 9;13:5312. doi: 10.1038/s41467-022-32738-7 (PMC9463451; doi:10.1038/s41467-022-32738-7)
Supplement: Supplementary file 3 — Description of Additional Supplementary Files [file 41467_2022_32738_MOESM3_ESM.docx]

**Description of Additional Supplementary Files**

File Name: Supplementary Movie 1: Visualizing blood flow and leukocyte trafficking inside blood vessels

Description: This video demonstrates blood flowing inside blood vessels (red annotated areas) in real-time found within dermis of a patient with a skin lesion. Few leukocytes seem to slow down along the edges of the endothelium, which implies participation in the leukocyte trafficking cascade.

File Name: Supplementary Movie 2: Leukocyte rolling and adhesion inside blood vessel

Description: This video demonstrates a blood vessel with leukocytes that are temporarily adherent (top endothelium) or rolling (red arrow) along the endothelial wall.

File Name: Supplementary Movie 3: Leukocyte crawling inside blood vessel

Description: This video demonstrates a blood vessel with leukocytes that are inching or crawling (red arrow) along the endothelial wall.
