# Supplementary material for: In vivo tumor immune microenvironment phenotypes correlate with inflammation and vasculature to predict immunotherapy response
Source: Nat Commun. 2022 Sep 9;13:5312. doi: 10.1038/s41467-022-32738-7 (PMC9463451; doi:10.1038/s41467-022-32738-7)
Supplement: Supplementary file 8 — Source Data [file 41467_2022_32738_MOESM8_ESM.zip › source data/Source Data Information.docx]

**Source Data Information**

Source data has been provided for Figures 2, 3 4, 5, 6 and 7 (including Figures S3, S4, S5, S6 and S7) and included in a zipped folder.

**Figure 2:**

- Excel sheet containing RCM evaluation from 27 BCC lesions used for HCPC and PCA in figure 2.

**Figure 3:**

- Cpmnormlog2_filtergeneid_inputcemi.txt file was used as input for generating gene expression modules using CEMiTool. Supplemental Figure 2c-e and Figure 3a-e as well as Supplemental Figure 3a-f were generated using this input gene expression data.
- Modulegenes.tsv is a list of genes and respective module assignment resulting from CEMiTool analysis
- Zipped folder for GO enrichment using enrichr for module 2 and 5 associated with figure 3c and S3e
- CIBERSORTx_Job12_Results.xlsx CIBERSORTx output estimating cell proportions for each sample used to generate plots for Figure 3f and h.
- LM22.txt reports genes used to estimate cell proportions resulting from CIBERSORTx. Differential expression of these transcripts across samples were used to generate plot in Figure 3g.
- Zipped files in TissueNexusInteraction were used to generate module gene interactions for specific tissues/cell type (blood.txt, macrophage.txt, skin.txt, t_lymphocyte.txt) displayed in Figure 3d and Supplemental Figure S3g. Gene expression for module hub genes along with network hub genes (intermod2hubtidy.txt and intermod5hubtidy.txt) were shown in Figure 3e.

**Supplemental Figure 3:**

- edgeRDEGfullbulk_pairwiseresults.txt files reports the results from pairwise comparison of differential gene expression analysis using edgeR. logFC, average logCPM expression, PValue, FDR, and gene ensemble id as well as gene symbol are reported. Supplemental Figure 3g MA plot was generated using this output.

**Figure 4 and Supplemental Figure 4:**

- Sheet 4a contains the multiplexed IF analysis values, including raw counts and calculated positive cell counts in both intratumoral and peritumoral regions in BCCs that were used to generate column scatter plots showing distribution of cells across the three phenotypes in figure 4a and supplementary figure S4a
- Sheet 4b contains the calculated CD3^+^ CD20^+^ positive and TLS areas in BCC specimens that were used to generate the column scatter plots showing distribution of cell and TLS positivity across the three phenotypes in figure 4b

**Figure 5 and Supplemental Figure 5:**

- Sheet 5a contains RCM evaluation from 13 melanoma lesions used for HCPC and PCA analysis in figure 5a
- Sheet 5b contains calculated CD3^+^ T-cell area positivity and TLS area in melanoma specimens that were that were used to generate the column scatter plots showing distribution of cell and TLS positivity across the two phenotypes in figure 5b

**Figure 6:**

- Sheet 6a contains the quantified RCM values for vessel diameter, count and area, vessel trafficking counts, total inflammation density and only leukocyte-like density along with corresponding gene expression values used for correlation and plotted in figure 6a. The RCM values were also used in correlation analysis with module eigenvalues in figure 6b.
- Sheet 6b the eigen value for each module from CEMiTool which was used to correlate with RCM TiME traits; module 5 sig correlated with infiltrating myeloid shown in Figure 6b.

**Supplemental Figure 6:**

- Sheet S6 reports results from Spearman correlation of M2 and M5 eigengene values with RCM TiME phenotypes. Correlation and pvalue for each relationship is reported and represented as correlation matrix in Figure S6f and scatterplot of M5 eigenvalue against infiltrating myeloid cells in Figure 6b

**Figure 7 and Supplemental Figure 7:**

- Sheet 7a lists detailed RCM evaluation for imiquimod responders (R) and non-responders (NR) used to create HCPC clusters in figure 7a and heatmap, plots in figure 7c. These evaluations were used for linear regression modeling in figure S7.
- Sheet 7b lists RCM evaluation for key features from original analysis on 27 BCC lesions and 13 imiquimod treated lesions to predict phenotype in figure 7b
