# Supplementary material for: In vivo tumor immune microenvironment phenotypes correlate with inflammation and vasculature to predict immunotherapy response
Source: Nat Commun. 2022 Sep 9;13:5312. doi: 10.1038/s41467-022-32738-7 (PMC9463451; doi:10.1038/s41467-022-32738-7)
Supplement: Supplementary file 8 — Source Data [file 41467_2022_32738_MOESM8_ESM.zip › source data/Figure 3_S3/SourceDataDescription.docx]

**Source Data**

**Figure 3:**

- Cpmnormlog2_filtergeneid_inputcemi.txt file was used as input for generating gene expression modules using CEMiTool. Supplemental Figure 2c-e and Figure 3a-e as well as Supplemental Figure 3a-f were generated using this input gene expression data.
- Modulegenes.tsv is a list of genes and respective module assignment resulting from CEMiTool analysis
- Zipped folder for GO enrichment using enrichr for module 2 and 5 associated with figure 3c and S3e
- CIBERSORTx_Job12_Results.xlsx CIBERSORTx output estimating cell proportions for each sample used to generate plots for Figure 3f and h.
- LM22.txt reports genes used to estimate cell proportions resulting from CIBERSORTx. Differential expression of these transcripts across samples were used to generate plot in Figure 3g.
- Zipped files in TissueNexusInteraction were used to generate module gene interactions for specific tissues/cell type (blood.txt, macrophage.txt, skin.txt, t_lymphocyte.txt) displayed in Figure 3d and Supplemental Figure S3g. Gene expression for module hub genes along with network hub genes (intermod2hubtidy.txt and intermod5hubtidy.txt) were shown in Figure 3e.

**Supplemental Figure 3:**

- edgeRDEGfullbulk_pairwiseresults.txt files reports the results from pairwise comparison of differential gene expression analysis using edgeR. logFC, average logCPM expression, PValue, FDR, and gene ensemble id as well as gene symbol are reported. Supplemental Figure 3g MA plot was generated using this output.
